# Transcriptome profiling revealed novel transcriptional regulators in maize responses to *Ostrinia furnacalis* and jasmonic acid

**DOI:** 10.1371/journal.pone.0177739

**Published:** 2017-05-16

**Authors:** Hai Wang, Shengyan Li, Shouzhen Teng, Haisheng Liang, Hongjia Xin, Hongjiang Gao, Dafang Huang, Zhihong Lang

**Affiliations:** Biotechnology Research Institute, Chinese Academy of Agricultural Sciences, Beijing, P. R. China; Estacion Experimental del Zaidin, SPAIN

## Abstract

Chewing insects cause severe yield losses in crop production worldwide. Crop plants counteract chewing insects by transcriptionally promoting a repertoire of defense gene products that are either toxic to, or attractive to the natural enemies of, pest insects. However, the complexity of the transcriptional reprogramming in plant defense response against chewing insects is still not well understood. In this study, the genome-wide early responses in maize seedlings to Asian corn borer (ACB, *Ostrinia furnacalis*) and also to jasmonic acid(JA), the pivotal phytohormone controlling plant defense response against herbivory, were transcriptionally profiled by RNA-Seq. Clustering of differentially expressed genes (DEGs) along with functional enrichment analysis revealed important biological processes regulated in response to ACB infestation and/or jasmonic acid. Moreover, DEGs with distinct expression patterns were differentially enriched with diverse families of cis-elements on their promoters. Multiple inventories of differentially expressed transcription factors (DETFs) in each DEG group were also analyzed. A transient expression assay using transfected maize protoplastswas established to examine the potential roles of DETFs in maize defense response and JA signaling, and this was used to show that *ZmNAC60*, an ACB- and JA-inducible DETF, represented a novel positive regulator of JA and defense pathway genes. This study provided a comprehensive transcriptional picture for the early dynamics of maize defense responses and JA signaling, and the identification of DETFs offered potential targets for further functional genomics investigation of master regulators in maize defense responses against herbivory.

## Introduction

The plant defense response against chewing insects is bipartite [1]. One branch of the defense system is termed direct defense, in which plants produce toxins to fight herbivores. These toxins can be proteinaceous, such as the protease Mir1-CP [2, 3], ribosome-inactivating proteins (RIPs) [4], and protease inhibitors [5]. They can also be secondary metabolites, such as DIMBOA [6], glucosinolates [7], pyrrolizidine alkaloids [8], and phenolics [9]. In addition to direct defense, plants also evolved another layer of defense response termed indirect defense, in which plants produce volatiles to attract the natural enemies of herbivores [10, 11].

Most of the defense mechanisms mentioned above are absent or only present at basal levels in plants without herbivore attack, but strongly activated upon herbivore infestation [12]. This inherently requires coherent signal transmission and integration within plants for coordinated transcriptional reprogramming of numerous genes in direct or indirect defense responses. To achieve this, several critical steps are required, with the first step being perception of herbivory. Mechanical damage can be adopted by plants as a sign of herbivore invasion to induce the expression of several defense genes [13]. Plants also recognize herbivore-derived compounds termed herbivore-associated molecular patterns (HAMPs) with unknown receptors [14–18]. Perception of herbivory triggers a number of extreme-early downstream events, such as generation of reactive oxygen species and depolarization of cell membrane [19], calcium signaling [20], and activation of MAPK cascades [21]. These events collectively induce the biogenesis and mobilization of JA [22].

Among all phytohormones, JA plays the most pivotal role in the transcriptional reprogramming in plant defense response to herbivores. The importance of JA signaling in defense response against herbivory has been demonstrated by the fact that deficiency in JA biosynthesis or signaling leads to severely hampered defense response [23, 24]. At the transcriptome level, a large portion of transcriptional reprogramming occurred during mechanical damage or herbivory is mediated by JA-signaling [25, 26]. The central components in JA signaling have been identified and characterized mainly in model species *Arabidopsis* and rice, including *COI1*, *JAZ*, and transcription factors downstream of *JAZ* such as *MYC*s [27, 28]. In addition to *MYC*s, a large number of transcription factors were identified as crucial positive or negative regulators in JA-mediated defense response, such as *ORA59*[29], *ERF1* [30], *RIM1* [31], *bHLH3/13/14/17* [32], *JAM1/2/3* [33], *JAV1* [34], and *AtERF4* [35]. In another study, more than 40 transcription factors were found induced by *Spodoptera littoralis* in *Arabidopsis*, with at least 9 of them playing significant roles in resistance to *S*. *littoralis* herbivory [36].

Although the transcriptomic changes and transcriptional regulators of plant defense response against chewing insects have been extensively investigated in *Arabidopsis* and rice, less is known on the transcriptional control in maize herbivore resistance. Previous studies have investigated maize defense response against *Spodoptera frugiperda* [37], *Diabrotica undecimpunctata howardi* [38], and *Ostrinia furnacalis* [39]. In this study, the genome-wide early responses in maize seedlings to Asian corn borer (ACB, *Ostrinia furnacalis*) and JA, were transcriptionally profiled. A comparative transcriptome investigation was conducted, with emphasis on functional enrichment analysis, differential usage of exons, cis-element enrichment analysis, and differentially expressed transcription factors (DETFs). We also established a transient expression assay to examine potential roles of DETFs identified in this study in maize defense response and JA signaling, and this was used to show that *ZmNAC60*, an ACB- and JA-inducible DETF, represents a novel positive regulator of JA and defense pathway genes. This study provided a comprehensive transcriptional picture for the early dynamics of maize defense responses and JA signaling, and the identification of DETFs offered potential targets for further functional genomics investigation of master regulators in maize defense responses to herbivory.

## Materials and methods

### Plant materials, growth conditions, and treatments

B73 maize plants were grown in a growth room with 12 h light/12 h dark cycles at 24°C for 3 weeks until reaching the V3 stage. On the day of treatment, plants were randomly divided into three groups: Control group, *Ostrinia furnacalis* treatment group, and jasmonic acid treatment group, with each group containing 30 seedlings. The three groups were then moved into separate growth chambers with identical growth conditions, considering that jasmonate is highly volatile, and also that plant volatiles from one group may change gene expression in other groups. Treatments started 2 hours after the lights were on. For the herbivory treatment, three two instar larvae were placed into the whorl of each plant. For jasmonic acidtreatment, each plant was sprayed with a fine mist of 100 μM jasmonic acid (Sigma, USA) suspended in 5 ml distilled water. JA was sprayed on whole plants. Control and ACB-treated groups were also mock-treated with a fine mist of distilled water. Eight hours after the onset of the treatment, the third and forth leaves were harvested and frozen in liquid nitrogen for RNA-Seq analysis. Each treatment contained three biological replicates, with 10 plants pooled for each replicate. To test for systemic gene expression after mechanical wounding, maize seedlings were scratched with a razor blade once every hour across the midrib in the middle of the third leaves. To test for systemic gene expression after ACB damage, two two instar larvae were enclosed in a small cage made out of Eppendorf tube caps attached on the surface of the third leaves. Third or fourth leaves were harvested at indicated time points, with 3 biological replicates for each time point and 6 seedlings for each replicate.

### RNA-seq library construction, sequencing and analysis

RNA-seq libraries were generated from 5 μg total RNA and size-selected for 250–300 bp inserts for paired-end sequencing (100 bp for each end). Libraries were quantified on an Agilent bioanalyzer (Agilent, CA, USA) and sequenced using the Illumina HiSeq2000 system according to standard Illumina protocols (Illumina Inc., CA, USA). Raw reads were trimmed and filtered using Sickle (version 1.33). The Tophat2-Cufflinks pipeline [40] was employed in the updating of gene models in the RNA-Seq analysis. Reads were aligned to the maize reference genome (AGP v3.23) by Tophat2 (version 2.1.0), followed by optimization of existing gene models and also the identification of novel transcripts identified in this study using Cufflinks (version 2.2.1). Differentially expressed genes were identified using the HTSeq-DESeq pipeline [41]. Aligned reads were counted by HTSeq according to the updated genome annotation. Gene-level expression values were represented as Fragments per Kilobase per Million (FPKM). Differentially expressed genes were identified using the DESeq package [42]. A False Discovery Rate (FDR) corrected p-value≤0.05 and a threshold fold change≥2 was used to call differentially expressed genes. Differential usage of exons was detected using DEXSeq [43] with the criteria FDR≤0.05 and threshold fold change≥2. Principal component analysis (PCA) of samples were carried out by the prcomp function implemented in the stats package of R (version 3.3.1), and only the first two components were plotted. Hierarchical clustering of samples was carried out using the hclust function implemented in the stats package of R (version 3.3.1). To determine whether ACB and JA lead to similar or contrasting transcriptomic changes, differentially expressed genes (DEGs) in at least one of the two treatments were determined, followed by evaluation of their fold changes in response to each treatment. After that, the pearson correlation coefficient between the two treatments was calculated using ACB- and JA-induced fold changes of DEGs by the cor function implemented in the stats package of R (version 3.3.1).

### Quantitative RT–PCR

Total RNA was isolated using Trizol (Invitrogen, CA, USA), followed by first-strand cDNA synthesis using SuperScript III reverse transcriptase (Invitrogen, CA, USA). The primer sequences are listed in S1 Table. PCR was performed in a 25 μL reaction mixture with Maxima SYBR Green/ROX qPCR Master Mix (Invitrogen, cat. no. K0221) using the following program: 95°C for 3 min, 40 cycles of 95°C for 30 s, 55°C for 30 s, 72°C for 30 s, followed by one cycle of 95°C for 1 min, 55°C for 30 s and 95°C for 30 s. The instrument used for qPCR was the StepOnePlus system (Applied Biosystems, CA, USA). Target gene expression was normalized using EF1alpha as an internal control. Three biological replicates were included in quantitative RT-PCR analysis.

### Functional classification

Maize gene annotations using GO (Gene Ontology) terms were acquired using the BLAST2GO pipeline [44], combined with existent GO annotation retrieved from Gramene BioMart. Enriched GO terms were determined by using Fisher’s exact tests followed by Benjamini-Hochberg multiple-test correction.

### Cis-regulatory motif enrichment analysis

To determine enriched cis-regulatory elements on promoters of co-expressed genes, proximal promoter was defined as 2 kbp upstream and 500 bp downstream of transcription start site (TSS) since this region is adequate to capture the 5’UTR and the first intron of most maize gene models. A comprehensive collection of plant position weight matrices (PWMs) [45] and also PWMs deposited in the JASPAR database were used. Promoters were scanned for significantly enriched cis-elements using the PWMEnrich package in the Bioconductor project.

### Maize protoplast isolation and transient transactivation assay

Maize protoplast preparation and PEG-mediated transfection was performed as previously described [46]. A modified version of pCAMBIA3300, referred to as pCAMBIA3300m, was constructed by inserting into it a fragment containing an Ubiquitin promoter, a multiple cloning site, and a nos terminator. *ZmNAC60* CDS alone, or *ZmNAC60* fused at its C-terminus with SRDX (transcriptional repression domain) or VP16 (transcriptional activation domain) were cloned into pCAMBIA3300m. The constructs were transiently transformed into maize protoplasts. The transformed protoplasts were treated with or without 10 μM jasmonic acid for 3h before harvest. In order to normalize transformation efficiency, a construct containing GUS CDS driven by the CaMV 35S promoter was used as a transfection control reporter. RNA extraction, first-strand cDNA synthesis, and quantitative PCR were conducted as described above. Expression of marker genes was calculated using the conventional 2^−ΔΔCt^ method [47], followed by normalization by GUS activity for each sample.

## Results and discussion

### Establishment of experimental conditions to investigate the maize-*Ostrinia furnacalis* interaction

**Fig 1:**
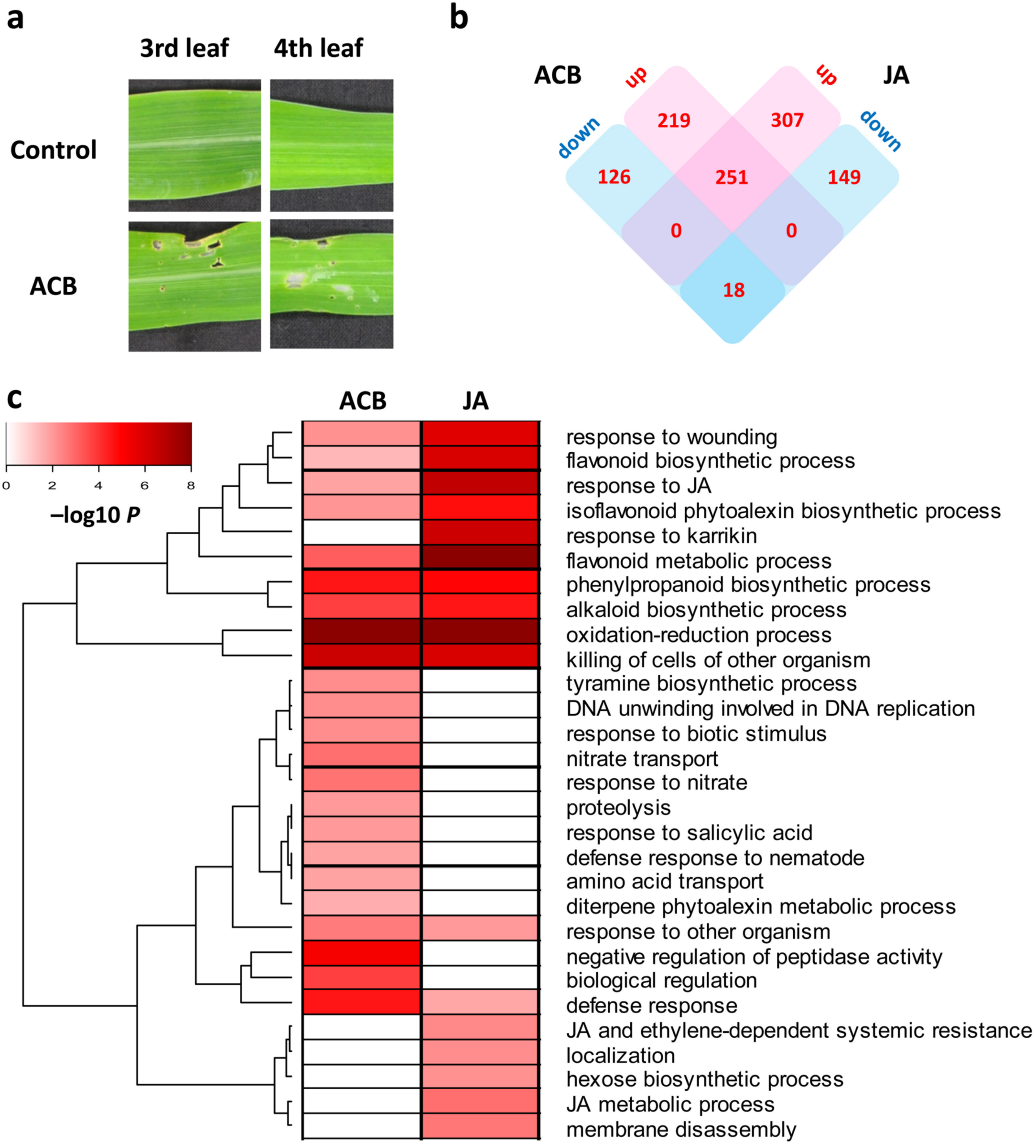
Dynamic reprogramming of maize transcriptome in response to ACB infestation and JA treatment. **a** V3 stage B73 maize seedlings were used in ACB treatment. After 8h ACB infestation, around 5% leaf surface were damaged. **b** A Venn diagram showing the number of DEGs with specific expression patterns in response to treatments. **c** A heatmap showing enriched biological processes in ACB- and JA-induced DEGs. Color was scaled based on log transformed false discovery rates, and darker colors indicated that the genes in the corresponding biological processes were more highly enriched in their respective DEG categories.

This study aimed to investigate the maize defense response to ACB. Maize plants were grown under a controlled environment in the growth chamber since numerous biotic or abiotic factors influencing maize-ACB interaction in field conditions would confound proper interpretation of data. Early-season ACB attack occurs at early or midwhorl stage as young larvae begin feeding within the whorl. However, as whorl stage maize is too large to grow healthily in growth chambers, we used three-week-old B73 seedlings. The third and forth leaves, which are preferred by ACB compared to the other two leaves, were harvested at various time points. The expression of a marker gene of ACB attack, *maize proteinase inhibitor* (*mpi*) [48], was monitored. The expression level of *mpi* was found significantly induced within 1h and reached steady state level at 8h (S1a Fig). At 8 h, around 5% of leaf surface, mostly distributed at leaf base area, was destructed (Fig 1a). Longer ACB attack caused extensive destruction of xylem and wilting of leaves, thus samples were harvested at 8h for RNA-Seq analysis. To conduct a comparative transcriptome analysis for ACB treatment and JA treatment, the expression of *allene oxide synthase1* (*aos1*), a marker gene for maize JA signaling [49], was monitored on JA-treated seedlings. Expression of *aos1* peaked at 6h and gradually decreased afterward (S1b Fig), suggesting that samples harvested at 8h were adequate to capture JA-induced gene expression. Another reason for simultaneous tissue harvest for control, ACB- and JA-treated samples was to eliminate circadian rhythm-caused differences in gene expression.

### ACB attack and JA treatment triggered distinct yet overlapping transcriptional reprogramming

To analyze the dynamics of the maize response to ACB and JA, global gene expression profiles upon 8h exposure to each treatment were inspected. The number of clean reads and mapping rates were listed in S2 Table. The Tophat2-Cufflinks pipeline [40] was employed to optimize existing gene models in the v3.23 genome annotation (S3 Table), and also to identify new gene models (S4 Table). Gene expression values in FPKM were based on the updated genome annotation. Principal component analysis (PCA) and hierarchical clustering showed high degree of reproducibility among biological replicates within each group (S2 Fig). A total of 614 genes were identified as differentially regulated in response to ACB damage (absolute fold change ≥2 and FDR adjusted p-value ≤0.05), with 470 genes up-regulated and 144 down-regulated. Comparable numbers of DEGs were found in JA-induced responses, with 558 genes up-regulated and 167 down-regulated (Fig 1b). Due to partial overlap between the two DEG sets, in total 1070 DEGs early-responsive to ACB and/or JA were observed, representing 2.7% of all maize genes (S5 Table). We observed a high correlation between ACB- and JA-induced transcriptomic changes (R^2^ = 0.73), indicating that JA-signaling indeed played a significant role in maize-ACB interaction. Validation of the expression levels of 20 genes by qRT-PCR showed strong correlation (R^2^ = 0.92) between results from RNA-seq and qRT-PCR (S1 Table).

To gain an insight into the biological processes involved in maize response to ACB and JA, over-represented GO (Gene Ontology) terms were analyzed for DEGs in each treatment. As shown in Fig 1c, Several GO terms were over-represented in both treatments, including 'response to wounding', 'response to JA', and also terms related to flavonoid, isoflavonoid phytoalexin, phenylpropanoid, and alkaloid biosynthesis, suggesting that biosynthesis of secondary metabolites in maize direct defense response was largely mediated by JA. Several GO terms were found only enriched in ACB treatment, but not in JA treatment. Examples include the GO term 'response to biotic stimulus' containing multiple orthologs of *Arabidopsispathogenesis-related* genes (*PR1*, *PR4*, *PR5*, and *PR10*), and 'response to salicylic acid', indicating that salicylic acid played important regulatory roles on genes that were ACB-inducible but JA-unresponsive. GO terms in this catogory also included 'negative regulation of peptidase activity' (mostly protease inhibitors) and 'diterpene phytoalexin metabolic process' (mostly terpene synthases), suggesting that induction of these genes mainly depends on signaling events other than JA pathway. Notably, there were also several GO terms specifically enriched in JA treatment, but not ACB treatment (Fig 1c), such as ‘JA metabolic process’ and‘JA and ethylene-dependent systemic resistance’, suggesting that corresponding genes might not be required for ACB resistance.

**Fig 2:**
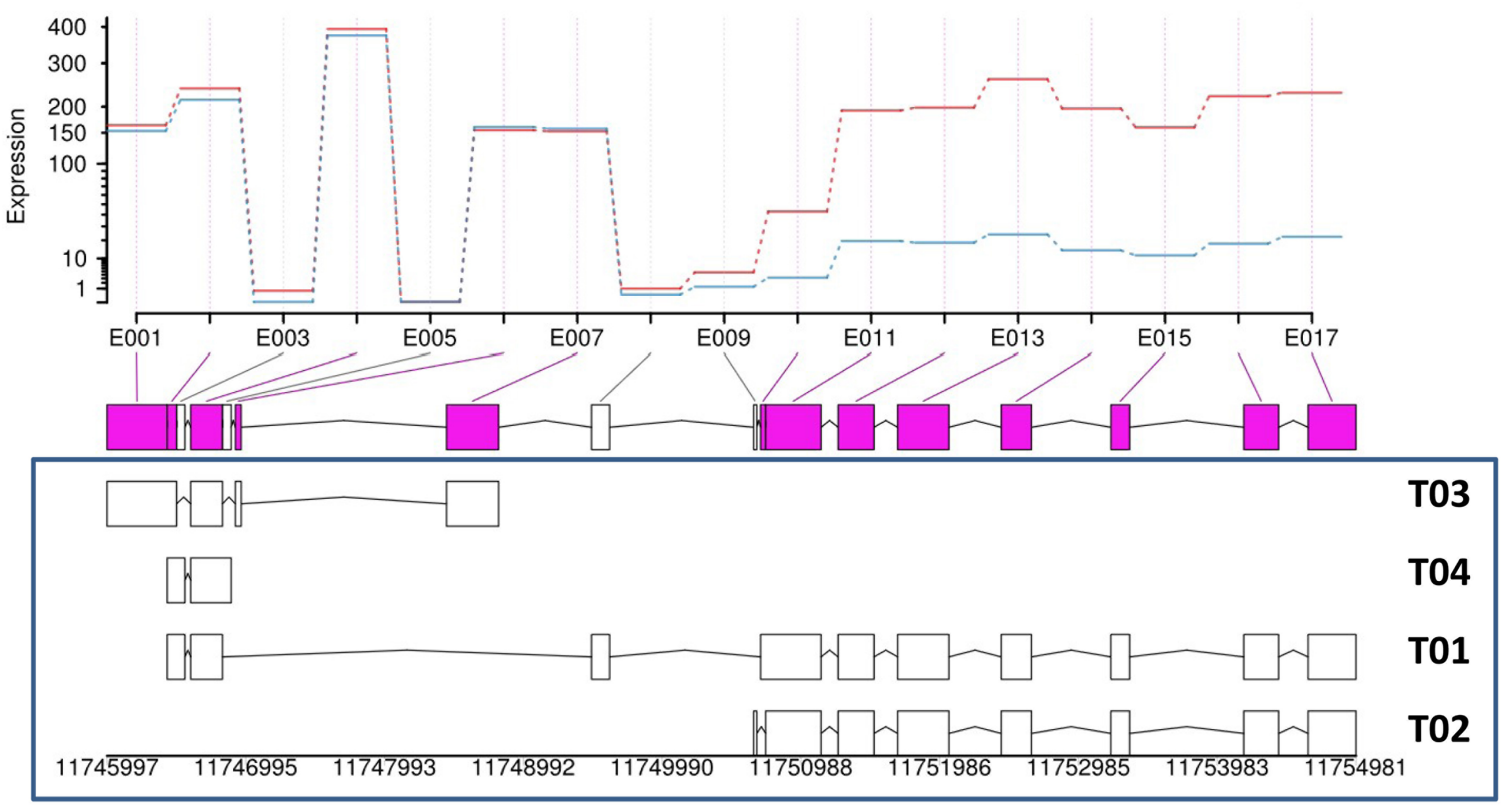
Differential usage of exons in *stc1* in response to JA treatment. The lower panel is a schematic representation of the four isoforms (T01~T04) of *stc1*. The middle panel is a collapsed view of all exons from the four isoforms. The upper panel represents mean expression levels of each exon in control (blue lines) and JA-treated (red lines) samples.

Alternative splicing has been reported to cause functional consequences. For example, production of JAZ repressors that are more stable at the protein level by alternative splicing prevents hyperactivation of the JA response in *Arabidopsis* [50]. In this study, differentially used exons (DUEs) in response to ACB and/or JA treatment were examined using DEXSeq. In total 16 DUEs from 8 genes were identified (S6 Table). Alternative usage of exons was not detected in any maize *JAZ* genes, suggesting that maize may adopt strategies different from that of *Arabidopsis* to control hyperactivation of the JA pathway. Notably, among the 16 DUEs, 8 were in gene *GRMZM2G177098*, which encodes *sesquiterpene cyclase1* (*stc1*), a maize gene that responds to caterpillar herbivory by producing a chemical defense signal to attract natural enemies of the herbivore [15]. There are four alternative splicing variants for *stc1* (designated T01~T04), and the elevated usage of the last 8 exons of *stc1* in response to JA treatment indicated preferential induction of T02, a shorter version of the full-length *stc1* (Fig 2). It would be interesting to investigate whether differential usage of exons of *stc1* would cause biochemical or physiological consequences.

### Differentially enriched cis-elements in distinct ACB- and JA-regulated gene clusters

**Fig 3:**
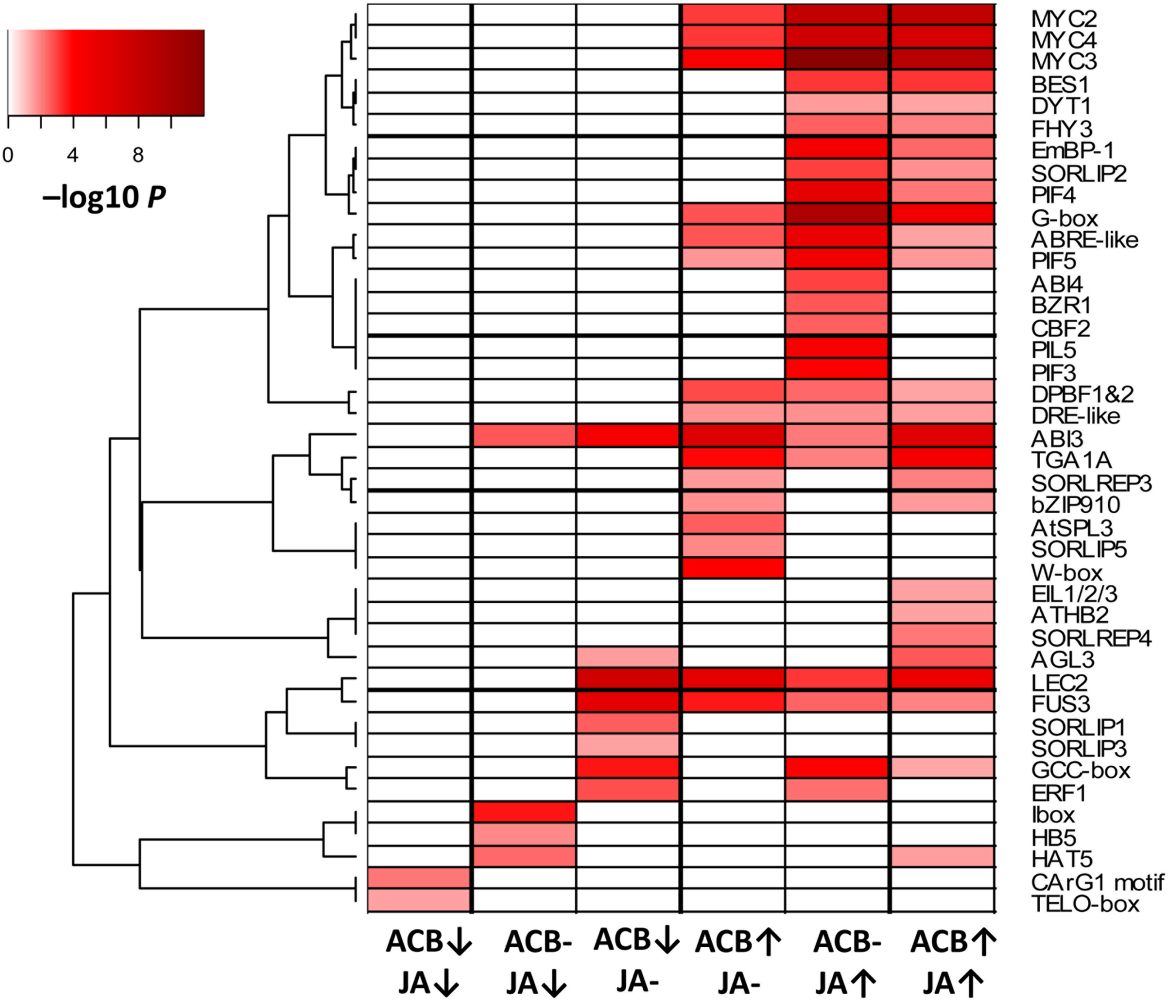
Enriched cis-elements in promoters of each DEG group. DEGs were grouped based on their expression pattern shown in Fig 1b. Cis-elements over-represented in at least one DEG group promoters were listed. Color was scaled to represent log transformed false discovery rates.

The 1070 DEGs were categorized into 6 groups according to their expression patterns shown in the Venn diagram in Fig 1b. The 6 groups were named ACB-up/JA-up, ACB-up/JA-normal, ACB-normal/JA-up, ACB-down/JA-down, ACB-down/JA-normal, and ACB-normal/JA-down, respectively. Genes with similar expression patterns generally share common regulatory cis-elements in their promoters, thus the enrichment of cis-elements for each DEG group was analyzed. This led to the identification of a total of 41 cis-elements enriched in at least one DEG group (Fig 3). Notably, the ACB-up/JA-up, ACB-normal/JA-up, and ACB-up/JA-normal group, although containing mutually exclusive sets of DEGs, shared a large number of enriched common cis-elements in their promoters, including MYC2/3/4 binding sites, G-box, ABRE-like motif, PIF5 binding site, DPBF1&2 motif, DRE-like motif, and also binding motifs for ABI3, TGA1A, LEC2 and FUS3. MYC2/3/4 are among the most pivotal master regulators in JA-signaling [51, 52]. ABRE-like elements, DRE-like elements, and DPBF1&2 binding sites were reported to be responsible for ABA and drought-responsive gene regulation [53–55]. Enrichment of these motifs implied an important role of ABA-signaling in maize response to ACB and/or JA. Moreover, over-representation of TGA1A binding sites, which were important for pathogen resistance responses [56], was consistent with enrichment of pathogenesis-related genes in our DEG sets. LEC2, FUS3 and ABI3 were reported to coordinately control biosynthesis of storage compounds including storage proteins and fatty acids [57]. In addition, A number of cis-elements were found only enriched in one or two of the ACB-up/JA-up, ACB-normal/JA-up, and ACB-up/JA-normal group, such as EIL1/2/3, BES1, BZR1, PIF3/4 binding sites, suggesting involvement of ethylene, BR and GA signaling in maize ACB and/or JA response. Interestingly, for ACB-down/JA-down, ACB-down/JA-normal, and ACB-normal/JA-down groups, cis-elements involved in plant development and light signaling were enriched, such as TELO-box, CArG1 motif, SORLIP1, SORLIP3, HAT5, and HB-5 binding sites. TELO-boxes were found enriched in the 5’ region of *Arabidopsis* genes encoding components of the translational apparatus [58, 59]. CArG1 box have been identified as targets of MADS domain transcription factors required for normal plant development [60–62]. These results suggested that corresponding transcription factors may play suppressive roles on their downstream target genes in maize herbivore defense response and JA response.

### Distinct inventories of transcription factors in ACB and JA response

**Fig 4:**
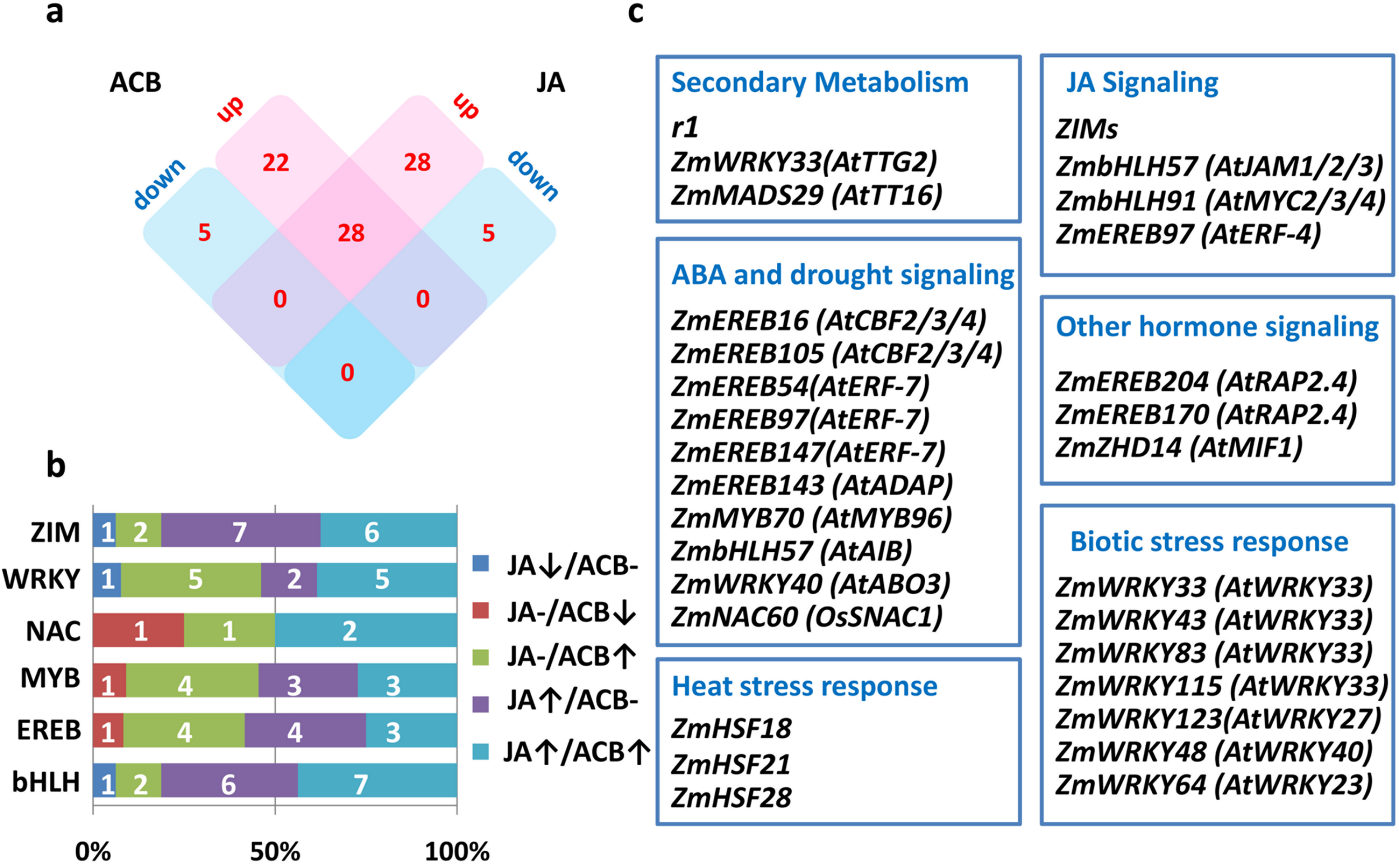
Transcription factors differentially regulated in response to ACB infestation and/or JA treatment. **a** A Venn diagram showing the number of DETFs in DEG groups defined in Fig 1b. **b** Distribution of TF families among DETF groups. **c** Representative functions and TFs differentially regulated in response to ACB and/or JA treatment. For clarity, functionally characterized *Arabidopsis* or rice orthologs were listed in parenthesis next to their maize counterparts.

Among all categories of DEGs, special attention was paid to TFs that are transcriptionally regulated, and are therefore potentially involved, in herbivore defense and JA signaling. To identify TFs in our DEG sets, DEGs with homology to known TFs were combined with information from the GRASSIUS database, followed by manual removal of mis-annotated genes in GRASSIUS, leading to the annotation of in total 88 TFs (summarized in S5 Table). Among the 614 ACB-responsive DEGs, 50 up-regulated and 5 down-regulated differentially expressed TFs (DETFs) were identified. 56 up-regulated and 5 down-regulated DETFs were identified in response to JA (Fig 4a). The two sets of DETFs partially overlapped (28 DETFs inducible by both ACB and JA treatments), a scenario observed for DEGs. The 88 DETFs belonged to 17 families, with only 6 families with more than 3 members. The distribution of the 6 families in different DEG groups was examined to identify family-specific expression trends (Fig 4b). We found that bHLH and ZIM family TFs largely belonged to the ACB-normal/JA-up group and ACB-up/JA-up group, while a larger proportion of WRKY, MYB and EREB family members belonged to the ACB-up/JA-normal group. In addition, transcription factors were absent in ACB-down/JA-down group.

DETFs identified in this study have not been functionally characterized, except for the *ZmbHLH1*/*r1*/*colored1* gene, a regulator of the anthocyanin pathway [63], thus putative functions of DETFs were assigned by their homology with characterized TFs in model species *Arabidopsis* or rice (summarized in S5 Table and Fig 4c). In addition to *r1*, *ZmWRKY33* and *ZmMADS29*, ortholog of *Arabidopsis TRANSPARENT TESTA GLABRA 2* (*TTG2*) [64] and *TRANSPARENT TESTA16* (*TT16*) [65], respectively, were potential regulators of proanthocyanidin biosynthesis in maize defense and JA responses. Notably, TFs involved in phytohormone-signaling were particularly enriched, especially JA and ABA signaling. JA-related TFs included 16 JAZ/ZIM family genes, *ZmbHLH57* (an ortholog of *ArabidopsisJAM1*/*2*/*3* [33]), *ZmbHLH91* (an ortholog of *Arabidopsis MYC2*/*3*/*4* [51, 52]), and *ZmEREB97* (orthologous to *ATERF-4*, a negative regulator of JA-responsive defense gene expression [66]). DETFs potentially involved in ABA signaling and drought stress response include: two orthologs of *CBF2*/*3*/*4* [67] (*ZmEREB105* and *ZmEREB16*), three orthologs of *ArabidopsisAtERF-7* [68] (*ZmEREB54*, *ZmEREB97*, *ZmEREB147*), *ZmMYB70* orthologous to *AtMYB96* [69], *ZmEREB143* orthologous to *ArabidopsisADAP* [70], *ZmbHLH57* orthologous to *Arabidopsis ABA-inducible BHLH-type transcription factor* (*AIB*) [71], *ZmWRKY40* orthologous to *Arabidopsis ABA overly sensitive mutant 3* (*ABO3*) [72], and *ZmNAC60* orthologous to rice *SNAC1* [73]. In addition to JA and ABA signaling, a number of DETFs were implicated in other phytohormone signaling as well, including *ZmEREB204* and *ZmEREB170*, two orthologs of *ArabidopsisRelated to AP2*.*4* (*RAP2*.*4*) that mediates light and ethylene signaling [74]; and *ZmZHD14*, an ortholog of *Arabidopsis Mini zinc Finger 1* (*MIF1*) that mediates GA signaling [75]. There were also a large number of DETFs implicated in biotic or abiotic stress responses. Four orthologs of *AtWRKY33* (*ZmWRKY83*, *ZmWRKY33*, *ZmWRKY115* and *ZmWRKY43*), an ortholog of *AtWRKY27* (*ZmWRKY123*), and an ortholog of *AtWRKY40* (*ZmWRKY48*), were in the DETF list. *AtWRKY33*, *AtWRKY27*, and *AtWRKY40* have been previously reported to regulate defense response against bacteria or fungi pathogens [76–78]. We also identified *ZmWRKY64*, an ortholog of *AtWRKY23* that was involved in nematode feeding site establishment [79]. DETFs potentially involved in abiotic stress responses include *ZmHSF18*, *ZmHSF28* and *ZmHSF21* belonging to the heat shock transcription factor family.

### *ZmNAC60* was a novel positive regulator of maize JA signaling

Among all DETFs identified in this study, we were particularly interested in *ZmNAC60*, since (1) *ZmNAC60* was among the most highly ACB- and JA-inducible genes; (2) Phylogenetic analysis of NAC family members from maize, rice and *Arabidopsis* revealed that *ZmNAC60* belonged to the SNAC subfamily (S3 Fig), according to the classification of NAC genes in rice [80, 81], and the role of SNAC family members in plant defense response against herbivores has not been defined, although they have been implicated in diverse abiotic and biotic stress responses in *Arabidopsis*, rice and poplar [26, 82–84]. Interestingly, *ZmNAC60* is on a small branch of the SNAC subfamily devoid of any *Arabidopsis* NAC genes, and the *Arabidopsis* NAC genes most closely related to *ZmNAC60* are *ATAF1/2*, which do not share sequence similarity with *ZmNAC60* outside the NAC domain (S3 Fig), suggesting that functionally equivalent counterparts of *ZmNAC60* might be absent in *Arabidopsis*. The rice NAC gene most closely related to *ZmNAC60* was *SNAC1*, a positive regulator of rice drought resistance [73, 85], and the role of *SNAC1* in defense response against herbivory was undefined.

**Fig 5:**
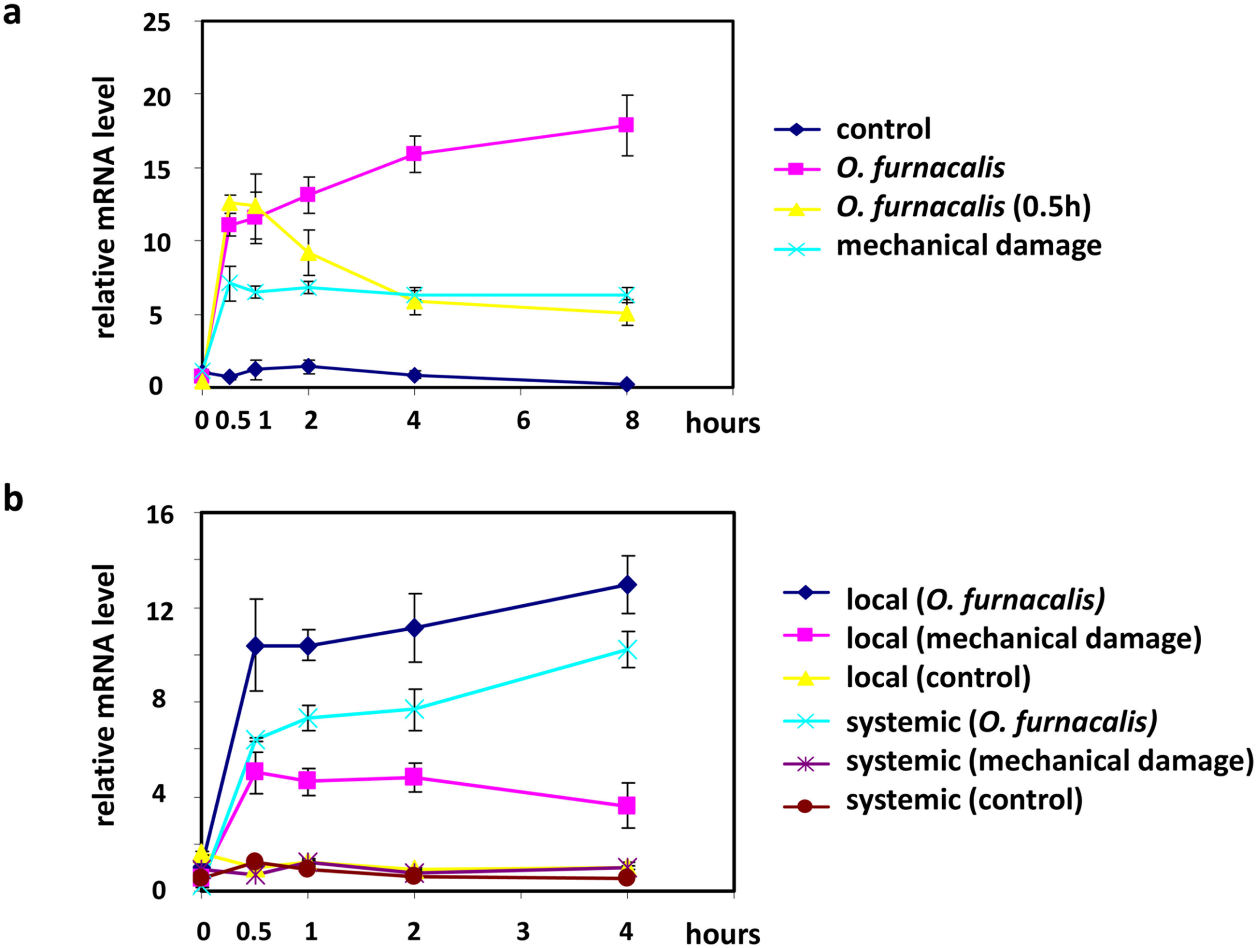
Time-series expression pattern of *ZmNAC60* in response to ACB attack and mechanical wounding. **a** Time-series expression pattern of *ZmNAC60* in maize leaves subjected to continuous ACB damage, mechanical wounding, or short-term ACB damage (0.5h treatment). Untreated maize plants were used as control. **b** The maize third leaves were subjected to ACB damage or mechanical wounding, and expression of *ZmNAC60* was quantified in the third (local tissue) as well as the forth leaves (systemic tissue). Error bars represent SEs from 3 biological replicates, with each replicate being a pooled sample containing leaves from 5~8 individual maize seedlings.

We first examined the expression patterns of *ZmNAC60* in response to ACB and mechanical wounding that mimicked ACB damage. As shown in Fig 5a, both ACB herbivory and mechanical wounding swiftly induced *ZmNAC60* expression within 0.5h, but ACB herbivory induced significantly higher levels of *ZmNAC60* expression, compared with mechanical wounding alone. Moreover, prolonged ACB treatment after 0.5h, but not mechanical wounding, continuously elevated *ZmNAC60* expression. These results indicated that mechanical wounding, albeit an important factor contributing to *ZmNAC60* induction, did not completely explain the expression pattern of *ZmNAC60*. In addition, when ACBs were removed from maize plants at 0.5h, the expression level of *ZmNAC60* decreased following ACB removal, indicating that hyper-accumulation of *ZmNAC60* transcripts was dependent on continuous ACB infestation. The signal of herbivory can be transmitted systemically in plants through multiple mechanisms [86, 87]. To investigate whether systemic herbivore signal would induce *ZmNAC60* expression, we restricted ACB damage and mechanical wounding on the third leaves of maize seedlings, and evaluated *ZmNAC60* expression in the forth leaves. As shown in Fig 5b, ACB damage induced *ZmNAC60* expression in systemic leaves, but the induction was not as strong as in local leaves. And mechanical damage failed to induce *ZmNAC60* expression in systemic leaves in our experiment.

**Fig 6:**
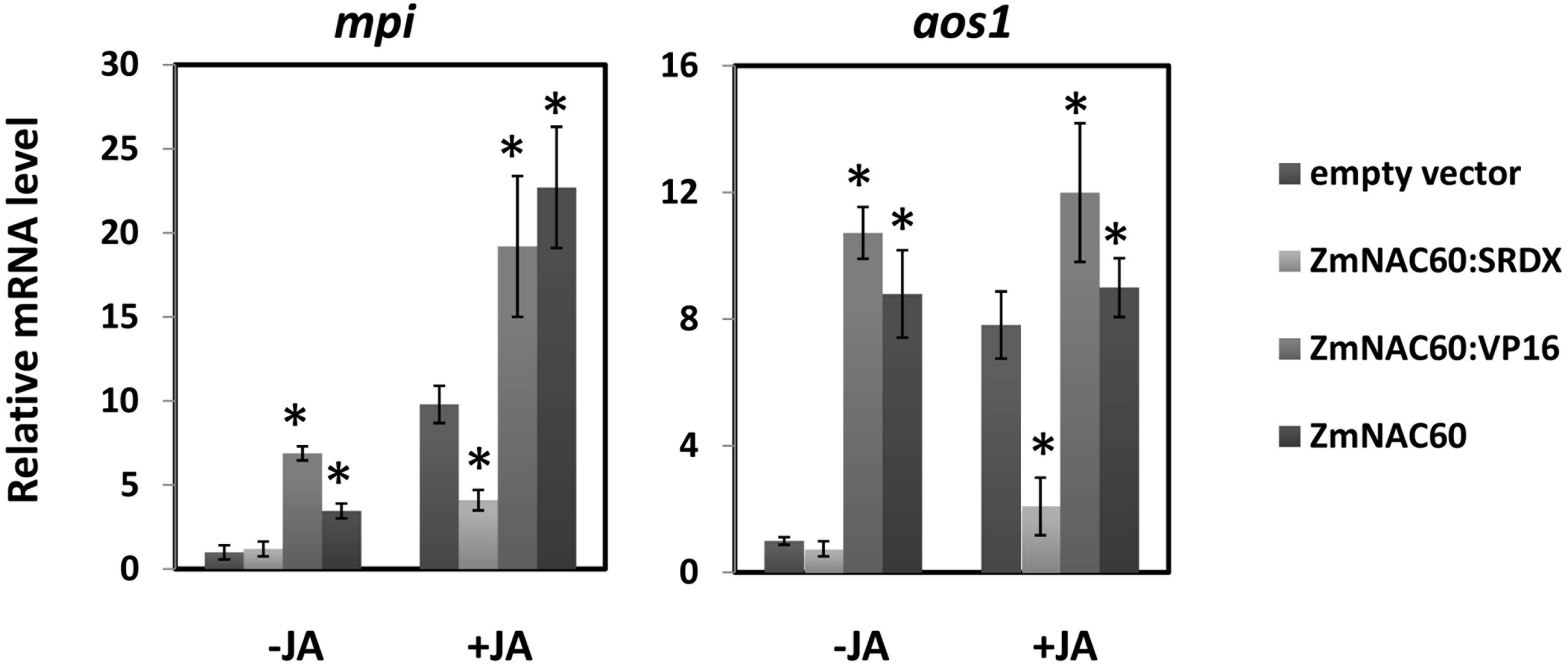
*ZmNAC60* transcriptionally regulated marker genes in maize defense response and JA signaling. ZmNAC60, ZmNAC60:SRDX, or ZmNAC60:VP16 were transiently expressed in maize protoplasts, driven by the constitutive Ubiquitin promoter, with empty vector used as control. The transiently transformed protoplasts were treated either with or without jasmonic acidfor 3 hours. Expression of *mpi* and *aos1* was quantified by qRT-PCR, and expression levels were normalized by transfection efficiency using a transfection control reporter vector constitutively expressing a GUS gene driven by the CaMV 35S promoter. Error bars represent SEs from 3 biological replicates. Student's t-test was used for comparisons between each sample with corresponding control sample transformed with the empty vector. * P < 0.05.

We then investigated whether *ZmNAC60* functioned as a transcriptional regulator in maize defense response and JA-signaling. The full-length ZmNAC60 protein, or ZmNAC60 fused with the transcriptional suppressor SRDX or transcriptional activator VP16, were transiently expressed in maize protoplasts driven by the constitutive Ubiquitin promoter (Ubi). The transiently transformed protoplasts were treated either with or without JA. The validity of this experiment system was confirmed by the fact that for protoplasts transformed with the empty vector, those treated with JA displayed significantly higher expression of *mpi* and *aos1* than those untreated with JA (Fig 6). In JA untreated groups, compared with protoplasts transformed with the empty vector, those transformed with Ubi::ZmNAC60:VP16 displayed higher expression of *mpi* and *aos1*, while the Ubi::ZmNAC60:SRDX construct did not significantly alter *mpi* and *aos1* expression. In JA treated samples, Ubi::ZmNAC60:VP16 and Ubi::ZmNAC60:SRDX transformation lead to significant induction and suppression of marker genes, respectively. Moreover, Ubi::ZmNAC60 displayed effects on marker gene expression similar to that of Ubi::ZmNAC60:VP16 (Fig 6). These results collectively indicated that ZmNAC60 and ZmNAC60:VP16 were positive regulators, while ZmNAC60:SRDX functioned as negative regulators of, *mpi* and *aos1* expression. Although it is still unknown whether *ZmNAC60* functions directly or indirectly upstream of *mpi* and *aos1*, we propose that this transient expression assay could be adopted to screen DETFs for novel regulators in maize defense response and JA signaling.

## Conclusions

In this study, a comparative analysis of transcriptome and cis-elements on ACB- and JA-treated maize revealed distinct yet overlapping signaling network for the two processes. Transcription factors responsive to ACB and/or JA treatments were identified, and a transient expression assay was established to screen for important regulators in maize ACB and JA response. As a proof of concept, we proved that *ZmNAC60* was a novel positive regulator for maize herbivore resistance response and JA signaling.

## Supporting information

S1 Fig Time-series expression pattern of *mpi* and *aos1* in ACB-treated and JA-treated maize, respectively. V3 stage B73 maize seedlings were treated with ACB or JA, and leaves were harvested at designated time points. Error bars represent SEs from 3 biological replicates, with each replicate being a pooled sample of 10 individual plants. Student's t-test was used for comparisons of treated and untreated (0h) samples. *P < 0.05.
(TIF)

S2 Fig PCA and hierarchical clustering of samples. (TIF)

S3 Fig Phylogenetic analysis of *ZmNAC60* and its homologs. The amino acid sequences of NAC domains of SNAC subfamily members were obtained from PlantTFDB and GRASSIUS. The evolutionary history was inferred using the UPGMA method implemented in MEGA6. The percentage of replicate trees in which the associated taxa clustered together in the bootstrap test (100 replicates) were shown next to the branches. The tree were drawn to scale, with branch lengths in the same units as those of the evolutionary distances used to infer the phylogenetic tree. The evolutionary distances were computed using the Poisson correction method and are in the units of the number of amino acid substitutions per site.
(TIF)

S1 Table Confirmation of RNA-Seq results by qRT-PCR. (XLSX)

S2 Table Statistics of reads mapped to maize reference genome. (XLSX)

S3 Table Optimization of gene models in the standard AGPv3.23 genome annotation. (XLSX)

S4 Table New genes identified in this study. (XLSX)

S5 Table A summary of expression levels and functional annotations of differentially expressed genes. (XLSX)

S6 Table A summary of differentially used exons in response to ACB infestation and JA treatment. (XLSX)
